# Family Perceptions of Newborn Cytomegalovirus Screening: A Qualitative Study

**DOI:** 10.3390/ijns7040080

**Published:** 2021-11-19

**Authors:** Michael J. Cannon, Denise M. Levis, Holly McBride, Danie Watson, Carol Rheaume, Mary Ann Kirkconnell Hall, Tatiana M. Lanzieri, Gail Demmler-Harrison

**Affiliations:** 1 National Center on Birth Defects and Developmental Disabilities, Centers for Disease Control and Prevention, Atlanta, GA 30333, USA; mrc7@cdc.gov (M.J.C.); DLevis@hrsa.gov (D.M.L.); 2 Department of Pediatrics, Baylor College of Medicine, Houston, TX 77030, USA; hollycorwin@gmail.com (H.M.); gdemmler@bcm.edu (G.D.-H.); 3 Kirby Marketing Solutions, Tampa, FL 33609, USA; dwatson@watsongroupmarketing.com (D.W.); carolerheaume@hotmail.com (C.R.); 4 Eagle Global Scientific for the National Center for Immunization and Respiratory Diseases, Centers for Disease Control and Prevention, Atlanta, GA 30333, USA; nmp8@cdc.gov; 5 National Center for Immunization and Respiratory Diseases, Centers for Disease Control and Prevention, Atlanta, GA 30333, USA

**Keywords:** congenital cytomegalovirus, newborn screening, parental perceptions, qualitative study

## Abstract

Objectives: We sought to understand long-term retrospective parental perceptions of the utility of newborn screening in a context where many affected children never develop sequelae but where intensive support services and ongoing healthcare were provided. Study design: Qualitative study. Methods: Focus groups and interviews among parents (N = 41) of children with congenital CMV who had been enrolled in a long-term follow-up study at a large medical college for a mean of 22 years following diagnosis. Groups included parents whose children were: symptomatic at birth; initially asymptomatic but later developed sensorineural hearing loss; and who remained asymptomatic into adulthood. Results: With proper follow-up support, newborn CMV screening was viewed positively by parents, who felt empowered by the knowledge, though parents often felt that they and healthcare providers needed more information on congenital CMV. Parents in all groups valued newborn CMV screening in the long term and believed it should be embedded within a comprehensive follow-up program. Conclusions: Despite initial distress, parents of CMV-positive children felt newborn CMV screening was a net positive. Mandatory or opt-out screening for conditions with variable presentations and treatment outcomes may be valuable in contexts where follow-up and care are readily available.

## 1. Introduction

Congenital cytomegalovirus (cCMV) infection occurs in 4.5 per 1000 live births in the United States and is a major cause of birth defects and developmental disabilities, including hearing loss and intellectual disability [1]. Some babies with cCMV infection exhibit symptoms at birth; others develop symptoms during childhood, but most—approximately 80%—never develop any symptoms [2]. Approximately 4000 US children each year develop disabilities due to cCMV infection. Symptomatic children may derive limited benefit from antiviral medications [3]. Initially asymptomatic children may benefit from early detection and intervention for hearing loss [4].

Newborn CMV screening is not part of the current recommended uniform panel [5,6]. Because most CMV-positive newborns never develop the disease, the utility of newborn CMV screening is likely to be especially sensitive to parental acceptability and psychosocial harms [7,8,9,10,11,12]. Parental attitudes have been assessed in hypothetical scenarios [13,14], but not among parents whose children were actually screened for cCMV in the real world. In this study, we assessed perceptions of the utility and acceptability of newborn CMV screening among parents of children who screened CMV positive or were diagnosed with cCMV and were followed through adolescence.

## 2. Methods

In September 2013, we conducted focus groups and interviews with parents of children with cCMV enrolled in a longitudinal study at a large medical college, hereafter referred to as the CMV Study. Children in the CMV Study participated in regular follow-up assessments at 4–6 weeks, 4–6 months, 9–12 months, 18–24 months, approximately yearly through elementary school, and then every 2–3 years through high school. Parents had regular consultations with cCMV experts. For children who were asymptomatic at birth, follow-up monitoring and evaluation in the CMV study were much more frequent and intensive than would be expected outside a research setting [15].

We recruited parents from three groups, defined by their child’s health outcomes at birth and during follow-up, and our study included a convenience sample of parents of children with cCMV enrolled in the CMV Study who agreed to participate in focus groups or interviews (Table 1). The Symptomatic Group consisted of 11 parents whose child was born symptomatic; all these children were identified through referrals rather than screening, and typically presented with hearing loss or intellectual disability. The Asymptomatic Late Sequelae Group consisted of nine parents whose child was born asymptomatic but who subsequently developed sequelae (typically sensorineural hearing loss). The Asymptomatic Group consisted of 19 parents whose child was born asymptomatic and who never developed disease sequelae.

Focus group and interview questions were similar across all three parent groups, though questions were modified to reflect experiences with disease sequelae in the Symptomatic and Asymptomatic Late Sequelae Groups. Questions included how parents felt when they received the positive newborn screen for CMV, probing on potential family and financial stressors; information needs after receiving the positive screen; benefits and challenges from receiving a positive screen; benefits and barriers encountered during the regular follow-up assessments; and opinions about whether and how congenital CMV screening should be offered to parents of newborns.

Two trained and experienced moderators facilitated all focus groups and in-depth interviews. All focus groups and most interviews were conducted in person; four interviews were conducted by phone. Focus groups lasted about 120 min and interviews lasted about 90 min. All focus groups and interviews were recorded, professionally transcribed, verified for accuracy, and imported into QSR NVivo© qualitative data analysis software. This research was approved by the institutional review board of the medical college affiliated with the CMV Study, and parents provided oral consent prior to participation.

Each transcript was thematically coded by two coders using inductive and deductive strategies to identify emergent themes. One coder was the focus group or interview moderator and the other was a researcher experienced with qualitative analysis who had not participated in the focus groups or interviews. The coding team assessed the newborn screening literature in order to situate the data within the larger context of the literature, and to check for possible coder biases by using other sources to verify categories and themes (Appendix A) [16] Coders regularly met to review and compare coding categories (Appendix B) to identify and resolve coding discrepancies, and modify definitions of thematic codes. The analysis examined similarities and differences in themes across parent groups.

## 3. Results

At the time of participants’ interview, children were a mean 23 years old (SD = 5.2) and had spent 22 years (SD = 3.7) in the CMV Study. While most participants were the mothers (77%), fathers, stepparents, and one grandmother also participated. Their education level varied, with 48% having college or graduate degrees.

### 3.1. Attitudes about Newborn CMV Screening

Parents in all groups valued newborn CMV screening and subsequent follow-up assessments, although reasons varied slightly across groups (Table 2). Knowing their child’s CMV-positive status at birth helped them know what to monitor, who to consult, what to read, and where to look for solutions to current or potential problems.

When asked if they were glad that their child had been screened for cCMV, the majority of Asymptomatic Group parents responded, “Yes … in the long run”, or similarly. Most commented that the CMV screening result shocked them and provoked anxiety, especially from uncertainty about the disease and its prognosis, yet they valued the knowledge. One parent simply stated, “Not knowing the truth doesn’t make it not true”. They valued the CMV Study follow-up assessments because they provided regular monitoring of their child.

Asymptomatic Late Sequelae Group parents expressed no negative views about CMV screening and commonly described the newborn screening as “priceless” and “invaluable”. Some recalled that at the time of diagnosis, they were grieved by “news no parent wants to hear,” but quickly realized the value of the cCMV diagnosis and the benefit to their child and themselves. It empowered them to know what was happening with their child and be better informed to make decisions and take actions to make their child’s life better. Without the cCMV diagnosis, they felt they could have faced a costly, lengthy, and emotional “diagnostic odyssey” to identify the cause of their child’s issues. Many parents could not imagine any reason why a parent would not want their newborn screened: “I cannot think of a downside of knowing”.

Symptomatic Group parents valued CMV screening as a tool to quickly identify the cause of the symptoms, know how to help their child, learn other symptoms to anticipate, and plan for the future. Though their children’s diagnosis was made following testing for cCMV based on clinical suspicion rather than from newborn screening, they too expressed no negative views of newborn screening and, despite the stress of receiving the initial diagnosis, felt it was “absolutely worth it” to know. For instance, one said, “Oh gosh, I think it’s invaluable. It’s priceless. It’s given us so many answers that we wouldn’t have known otherwise and so much information to arm ourselves with as we raise him”. Another parent said, “It was a blessing that they did that […] screening that I… knew nothing about, because from the get-go we had a support group, we had a road map, we had help”.

Focus group participants were presented with three scenarios for newborn CMV screening: mandatory screening; opt-out screening, where parents have to actively request not to have their child screened; and opt-in screening, where parents have to actively request to have their child screened. Most parents in all groups preferred mandatory screening, with three overarching themes: (1) cCMV is a dangerous infection; (2) early intervention is essential to improve health and developmental outcomes when a newborn tests positive; and (3) withholding this information from parents is wrong when cCMV can be so devastating to children and their families. However, opinions differed somewhat across the groups, with some seeing advantages to the opt-out or opt-in approaches.

Overall, most Asymptomatic Group parents were in favor of mandatory CMV screening. Concerns among Asymptomatic Group parents about mandatory screening included parents’ rights, state regulations, government interference and “nanny states”, as well as doing what is right for vulnerable groups and protecting pregnant women. Some parents raised the concern that mandatory screening would cause unneeded anxiety, given that the risk of sequelae is low for asymptomatic children. The few parents who supported the opt-out option believed that states should not tell families what to do and preferred that parents make the final choice about screening. Several parents also stipulated that screening should be done only if follow-up assessments and a treatment program, such as the CMV Study, could be offered.

Asymptomatic Late Sequelae Group parents were overwhelmingly in favor of mandatory screening because it would mean more babies would be tested and receive the help they needed. They preferred opt-out screening because many tests are already being performed at birth; seeking consent for a single test just after birth seemed like an imposition at a time when parents are already overwhelmed. They believed more babies would be tested and helped if parents had to actively opt out of screening.

Parents in the Symptomatic Group also overwhelmingly supported mandatory screening, expressing that early detection and intervention were essential to secure the best outcomes for children. One said that mandatory screening was justifiable because, “Number one, it’s noninvasive and, number two, there are treatments available for it, and there are options for the family”. The few Symptomatic Group parents who did not support mandatory screening were “not really big on government mandates” in general; however, these same parents felt that the benefits of newborn CMV screening outweighed drawbacks. Otherwise, the diagnosis or proper treatment of a vulnerable child could be delayed. During these discussions, many parents expressed distress about their lack of awareness about cCMV and that they had not been given a chance to prevent the infection in their child. Symptomatic Group parents spoke frequently about how mandatory screening could raise awareness about cCMV and how to prevent infection during pregnancy, thus protecting future parents and children from the “agony of what we went through”.

### 3.2. Impact of Receiving Initial CMV Test Results

The tone of parents’ emotional reactions to receiving the news of the CMV test results varied, but almost all reactions were negative (Table 3).

Many Asymptomatic Group parents discussed feeling unhappy and depressed about a baby who might develop significant health issues in the future. Many took months, or even years, to adjust to the cCMV diagnosis. Asymptomatic Late Sequelae Group parents reported feeling scared by the news of a disease they had never heard of. One mother said, “I was like, oh my gosh, this could be a lifelong challenge, this could be a game-changer for sure”.

Some Symptomatic Group parents also reported feeling emotional turmoil. One parent said, “I guess the age-old question is, ‘Why me?’ For the first couple of months, I blamed myself, my husband blamed himself”. Another said, “I was having emotional distress because of the fact that my child wasn’t ‘normal’”. Several mentioned that they received the initial test results just hours after giving birth, and because of their children’s acute symptoms, these parents were often isolated from their newborns just as these worries surfaced.

Parents in both the Asymptomatic Late Sequelae Group and the Symptomatic Group recalled having many questions about cCMV and felt frustrated when medical professionals did not have answers. Parents whose children were identified through referrals reported initially receiving poor medical information about cCMV. By contrast, parents whose children were identified through screening said that the high-quality medical advice they received calmed their fears.

### 3.3. Impact on Parents of Knowing Their Child Had cCMV

Parents spoke of their knowledge as two sides of the same coin, recalling that it promoted ongoing observation (as part of the CMV Study and at home) and a better understanding of their child’s development, as well as worry about their child’s uncertain future.

Among Asymptomatic Group parents, uncertainty and fear were prominent. One parent recalled, “It was always the ‘what if’ in the back of your mind. Is he going to develop? How is he going to do? Is he normal and plays with blocks?” Parents said the uncertainty and fear were strongest and more frequent when their child was an infant. One said, “He would be asleep and I’d sneak up to him and clap real loud to see if he will wake up, and a couple of times he didn’t and I would say, ‘Oh my God.’”

Asymptomatic Late Sequelae Group parents remembered feeling fear and uncertainty early on. In addition, they described feelings of panic when sequelae surfaced (most commonly hearing loss) and their child’s health changed quickly. One parent noted, “I got terrified. The last time he was [at the clinic], everything is fine and looked okay to us. Twelve months later, all his hearing in one ear has gone.” Parents reported that access to the CMV Study was “critical” as they navigated the uncertainties of their child’s future. They felt that information received through the CMV Study was very helpful as they advocated for their child within the school systems. One parent recalled that knowledge about her daughter’s hearing loss helped her convince the teacher to move her child’s seat so that she could better hear during class.

Symptomatic Group parents, whose children were already showing signs of cCMV disease at birth, expressed fear, uncertainty, and sadness about their child’s health. For some, the fear manifested as elevated vigilance. One participant described watching his daughter closely for any signs of health issues “to get her a jump on any sort of illness that she had…this is not a normal child. She does not have the means of communicating. When things go bad, they go bad quickly.”

Many Symptomatic Group parents characterized their knowledge about cCMV and access to follow-up assessments as “empowering.” Some expressed gratitude for the newborn CMV diagnosis and worried about parents of children with cCMV without a diagnosis: “I wonder how many [parents] out there don’t know that their child had congenital CMV, and they just don’t know. And they are dealing with all these dilemmas.”

For many parents, anxiety and fear were still present during regular follow-up assessments. Overall, however, they reported that they were better able to navigate the complexities of cCMV because participating in the CMV Study made them well informed of the potential health and developmental effects of the virus. A parent said, “It prepared me for what could happen. If I hadn’t known then, it would’ve been even more sad.”

## 4. Discussion

Our main findings were as follows: (1) positive newborn CMV screening results often caused stress and anxiety, sometimes at high levels, especially during the first notification; (2) stress and anxiety diminished over time; (3) parents were calmed by quality advice and follow-up care; (4) parents valued developmental screening and early interventions; and (5) parents valued newborn CMV screening and felt it should be offered for all newborns.

Overall, parents in all groups were glad their child was screened for (or in the case of the Symptomatic group, tested for) cCMV and supported universal newborn CMV screening. As expected, this support was strong among parents whose children had symptoms at birth or who developed late-onset sequelae. More surprisingly, parents of children who always remained asymptomatic also expressed strong support for newborn CMV screening. This support was reflected in a recent study among adult women, where knowledge levels of cCMV were low, but support for screening was very high when respondents were educated about the condition [14]. Parental attitudes towards screening are complex and change over time, demonstrating the challenges inherent in assessing the utility of newborn screening [17]. For conditions such as cCMV, where treatment benefits are modest and risks apparent, newborn screening with parental consent may be a more logical and ethical approach than mandatory screening [7,18,19].

One potential harm of newborn CMV screening is the risk of adverse effects from medical treatment of asymptomatic or mildly symptomatic infants who would not meet eligibility criteria for antiviral therapy and who would not have been diagnosed in the absence of screening [12]. Other potential harms include psychosocial and logistic burden on families from more frequent clinical evaluations (e.g., for early detection of sensorineural hearing loss), and disparities in access to diagnostic evaluation and antiviral therapy among infants who screen positive [12]. We found that parents whose children were identified through screening found the high-quality medical advice they received reassuring. All parent groups emphasized that appropriate education, support, and follow-up are crucial. As noted above, the families in our study had access to frequent follow-up and specialized care from physicians trained to work with children with cCMV, which were typically funded by the CMV study. This support, which parents considered pivotal to their children’s development and their own psychosocial adjustment, may be difficult to replicate outside of research settings. Thus, the experience of parents in our study cannot be generalized to that of parents whose children might be diagnosed with cCMV as part of a future newborn screening program.

Our study has important limitations. By virtue of their willingness to participate in the study, parents were likely to be more highly motivated to seek out answers and support, and accordingly be in favor of universal or opt-in testing, than members of the general public might be. Participants tended to be highly educated; it is unclear how results might have differed for parents with lower education levels. Another limitation of our study was the opportunity for recall bias, as parents described events that occurred years and even decades previously. However, participants had a long-term perspective to draw upon, which might have given them a more holistic view of the value of newborn CMV screening. Remarks made by this cohort of parents, specifically reactions to potential hearing loss or disability in general, may be bound somewhat by the time period in which the original screening and medical follow-ups were completed. Parental reactions to screening results and possible disability status, specifically hearing loss, may be different now as knowledge about interventions has changed over time. Finally, we did not interview parents whose children screened negative for cCMV.

Parents who were informed and whose children received supportive monitoring were strongly in favor of universal newborn CMV screening, even those whose children never developed disease sequelae. However, screening can introduce stress and anxiety among parents, especially immediately after diagnosis, a particular concern for cCMV since many children who screen positive will never experience disease sequelae, and possibly for other conditions in which disease manifestations may be extremely delayed in onset and variable in intensity. Such findings raise important considerations for policy deliberations about newborn CMV screening and the design of future screening programs, including systems for long-term follow-up and monitoring of children identified with cCMV as well as for support for their families.

## Figures and Tables

**Table 1:** Description of parent groups and types of information collection.

	Asymptomatic Group	Asymptomatic Late Sequelae Group	Symptomatic Group
Child health outcomes			
Birth	Asymptomatic	Asymptomatic	Symptomatic
Childhood	Asymptomatic	Symptomatic	Symptomatic
Qualitative method			
One-on-one interview	2	7	7
Dyad interview	1	2	2
Focus group	3 (4, 5, & 6 parents)	0	0
Total parents	19	11	11

**Table 2:** Parents’ Attitudes about Newborn CMV Screening.

Parents’ Attitudes about Newborn CMV Screening	Asymptomatic Group	Asymptomatic Late Sequelae Group	Symptomatic Group
**Positive Values of CMV Screening**			
I would do it again because knowledge is power.	X	X	X
It should be routine, especially for prevention for pregnant women.	X	X	X
We can treat early and improve your child’s life.	X	X	
Test and the foreknowledge it provides are worth the stress at hearing news “no parent wants to hear.”			X
Avoids a diagnostic odyssey, helps you move into solutions and action planning quickly.		X	
Spare other parents the agony we went through.			X
If positive, we’ll monitor your kid for health problems (caveat: if you have access to a program).	X	X	
Diagnosis made sure my child got services at school, college, and medically. Even disability accommodations for college testing.		X	
**Negative Values of CMV Screening**			
Anxiety provoking when the proportion of children who develop problems is so small.	X		
Confusion between value of CMV tests and 3-month hearing test.	X		
**Value of Follow-Up Testing**			
Helps you become a better parent by understanding child development milestones.	X	X	X
Helps parents be more attentive to possible learning or hearing disabilities and to address them as early as possible, helping your child to succeed in life.	X	X	X
Provides benchmarks for measuring progress, stabilization, or deterioration of child’s condition.		X	X
Permits early intervention for child development, language development, and hearing loss remedies.		X	X
**CMV Screening Options**			
If testing were routine, more babies would get tested and more babies would get help, provided a program is available.	X	X	
Make mandatory but parent should have the right to opt out of passive consent if they do not want their child to be tested.	X	X	
Mandatory testing should occur because innocent children are at risk.			X
Mandatory testing should occur for early intervention before it’s too late for the child’s health.			X

Xs indicate attitudes expressed by parent groups.

**Table 3:** Psychosocial Impacts on Parents.

Psychosocial Impacts on Parents	Asymptomatic Group	Asymptomatic Late Sequelae Group	Symptomatic Group
**Concerns about CMV Child’s Development**			
*Positive Impact*			
Pride in affected child’s attitude and successes	X	X	
Felt calmer or less scared	X	X	
Felt well informed about risks		X	
Calmed by good medical advice and treatment programs		X	
Little or no fear for child’s future well being		X	
Pride in meeting the challenges		X	
No major positive impacts			X
*Negative Impact*			
Felt scared or panicky	X	X	
Fear for child’s future well being		X	X
Fear for child’s future health			X
**Concerns about Child’s Future Physical Health Development**			
*Positive Impact*		None	
Felt well informed about risks	X		
Calmed by good medical advice	X		
Felt calmer or less scared			X
*Negative Impact*			
Fear for child’s future health	X	X	X
Felt scared or panicky	X	X	
Felt under strain			X
**Concerns about Managing the Educational System in Relation to Their CMV Child**			
*Positive Impact*	None	None	None
*Negative Impact*	None		
Concerns about how child would be accepted by family and friends		X	
Felt under strain			X
**Emotion of Receiving Initial CMV Test Results**			
*Positive Impact*	None	None	None
*Negative Impact*			
Felt scared or panicky	X	X	
Felt unhappy and depressed	X		
Felt under strain			X
**Employment Issues Related to CMV Child**			
*Positive Impact*	None	None	None
*Negative Impact*	None	None	
Felt under strain			X
**Family Issues**			
*Positive Impact*			
Improved social supports, both tangible and intangible	X	X	X
Unconcerned or little concern about how child would be accepted by family and friends		X	
Felt calmer or less scared		X	
*Negative Impact*		None	
Perceived reduced intangible social support	X		
Had strained relationships with family and friends			X
Felt under strain	X		
**Financial Issues**			
*Positive Impact*			
Unconcerned or little concern about healthcare costs of tests and treatments, if needed	X	X	
Felt calmer or less scared		X	X
*Negative Impact*		None	
Concern about healthcare costs of tests and treatments, if needed	X		X
**Friends and Their Social Support**			
*Positive Impact*	None	None	None
*Negative Impact*	None	None	None
**Marital Concerns and Issues**			
*Positive Impact*			
Improved relationships	X	X	X
*Negative Impact*	None	None	None
**CMV Medical Assistance and Testing Programs**			
*Positive Impact*			
Caring medical practice or system	X	X	X
Felt reassured by good medical advice, treatment programs	X	X	X
Felt well informed about risks	X		
Felt happy and encouraged	X		
Felt calmer or less scared	X		
Unconcerned or little concern about healthcare costs of tests and treatments, if needed		X	
*Negative Impact*		None	
Concerns about unnecessary medical visits	X		
Impersonal or uncaring medical practice or system (isolated and infrequent)			X
**Medical Practitioners and Systems Related to CMV Child**			
*Positive Impact*	None	None	None
*Negative Impact*			
Felt anger (or dissatisfaction) at poor medical advice outside CMV Program	X	X	X
**Parenting Skills**			
*Positive Impact*	None		
Feeling clear about what would be best for my child		X	
Pride in meeting the challenges			X
*Negative Impact*	None	None	
Concerns about properly caring for the child			X
**Parent-Child Relationship Related to CMV Child**			
*Positive Impact*			None
Unconcerned or little concern about parenting and parent-child bond	X	X	
*Negative Impact*	None	None	None

Xs indicate psychosocial impact expressed by parent groups. None indicates that psychosocial impact was not expressed by parent groups.

## Data Availability

The data presented in this study are available on request from the corresponding author Tatiana M. Lanzieri (tmlanzieri@cdc.gov). The data are not publicly available due to privacy restrictions.

## Appendix A

**Table A1:** Categories of Psychosocial Impacts Identified in Literature Review.

Literature Review Summary Categories of Psychosocial Impacts	# of Mentions
Felt nervous or strung up Felt scared or panicky	21
3. Concern about healthcare costs of initial test, follow up tests, and future treatment, if needed	11
4. Improved social support, both tangible and intangible	9
5. Concerns about properly caring for child	8
6. Anger from not knowing risks	6
7. Felt unhappy and depressed	7
8. Fear for child’s future health	8
9. Felt others were to blame for child’s health issues 10. Felt I was to blame for child’s health problems	6
11. Concerns about parenting and parent-child bond	6
12. Felt worried about the future 13. Fear for child’s future well being	5
14. Concerns about how child will be accepted by family and friends	5
15. Concern about unnecessary medical visits	5
16. A greater sense of well being 17. Pride in meeting the challenges	4
18. Pride in affected child’s attitude and successes	4
19. Anger at poor medical advice	4
20. Feeling confused about what will be best for my child	3
21. Fear of treatment procedures for my child	2
22. Improved relationships with friends or family 23. Getting on better with those around you	2
24. Guilt over not taking preventative actions or not knowing about preventative actions.	2
25. Concern that medical professionals don’t explain things well 26. Feeling confused about medical advice	2
27. Hopeful about the future	2
28. Fear of future parenthood	2
29. Felt under strain	1
30. Felt stress to heavily research the health issues through family, friends, books, and Internet	1

Experienced researchers coded for specific psychosocial impacts that were identified in the literature review, which resulted in 155 codes entered into a database. Similar or nearly similar concepts and ideas were grouped together resulting in a total of 30 relatively unique impacts. Because these 30 codes were a combination of positive and negative impacts, we created corollaries for each code so that each negative impact had a positive impact in case a parent expressed the opposite feeling during the focus groups or interviews. The final table of coded negative and positive impacts can be found in Appendix B (Table A2).

## Appendix B

**Table A2:** Corollaries of Negative and Positive Impacts.

Negative Impact	Positive Impact
Anger (or dissatisfaction) at poor medical advice	Calmed by good medical advice, treatment programs
Anger (or dissatisfaction) from not knowing risks	Felt well informed about risks
Felt scared or panicky or Felt nervous or strung up	Felt calmer or less scared (often due to help from medical advice)
Felt unhappy and depressed	Felt happy and encouraged
Concern about being unaware of disease *	Unconcerned or little concern about being unaware of disease
Concern about healthcare costs of tests and treatments, if needed	Unconcerned or little concern about healthcare costs of tests and treatments, if needed
Concern about unnecessary medical visits	Unconcerned or little concern about unnecessary medical visits
Concern that medical professionals don’t explain things well	Unconcerned or little concern that medical professionals don’t explain things well
Concerns about how child will be accepted by family and friends	Unconcerned or little concern about how child will be accepted by family and friends
Concerns about parent-child bond	Unconcerned or little concern about parenting and parent-child bond
Concerns about properly caring for child	Unconcerned or little concern about properly caring for child
Depressed about the future	Hopeful about the future
Dishonor we did not meet the challenges better	Pride in meeting the challenges
Fear for child’s future health	Little or no fear for child’s future health
Fear for child’s future well being	Little or no fear for child’s future well being
Fear of future parenthood	Little or no fear of future parenthood
Fear of treatment procedures for my child	Little or no fear of treatment procedures for my child
Feeling confused about medical advice	Feeling clear about medical advice
Feeling confused about what will be best for my child	Feeling clear about what will be best for my child
Felt I was to blame for child’s health problems	Felt I took good care of myself during my pregnancy
Felt nervous or strung up	Felt calm and collected
Felt others were to blame for child’s health issues	Felt no one was to blame for my child’s health problems
Felt stress to heavily research the health issues through family, friends, books, and Internet	Felt confident I was getting good medical information from the doctors
Felt under strain	Felt at peace
Felt worried about the future	Felt unworried about the future
Guilt over not taking preventative actions or not knowing about preventative actions.	Sense of surety that I took all of the precautions I knew about
Having trouble with those around you	Getting on better with those around you
Poor sense of well being	Greater sense of well being
Reduced social support, intangible	Improved social support just intangible
Reduced social support both tangible and intangible	Improved social support both tangible and intangible
Sadness about my affected child’s attitude and successes	Pride in affected child’s attitude and successes
Strained relationships with friends and family	Improved relationships with friends or family
Impersonal or uncaring medical practice or school system *	Caring medical practice or system
Concerns about multiple locations to visit for healthcare treatments or logistics of treatment appointments *	Unconcerned or little concern about multiple locations to visit for healthcare treatments or logistics of treatment appointments

* New impacts identified through our interviews that were not found in the literature review.

During the coding of the original research for this project, researchers used the 30 codes identified in the literature review to categorize the psychosocial impacts that parents expressed, and created corollaries for each code so that each negative impact had a positive impact in case a parent expressed the opposite feeling during the focus groups or interviews.
